# Key informant views on potential acceptability and feasibility of long-acting antiretroviral treatment for HIV in Kenya

**DOI:** 10.1186/s12879-024-09309-w

**Published:** 2024-04-19

**Authors:** Anne Kaggiah, Catherine N. Maina, John Kinuthia, Douglas Barthold, Brett Hauber, Jacinda Tran, Jane M. Simoni, Susan M. Graham

**Affiliations:** 1 Research and Programs Department, Kenyatta National Hospital, Nairobi, Kenya; 2 Department of Global Health, University of Washington, Seattle, WA USA; 3 The Comparative Health Outcomes, Policy, and Economics (CHOICE) Institute, University of Washington, Seattle, WA USA; 4 Worldwide Medical and Safety, Pfizer, Inc, New York, NY USA; 5 Office of Behavioral and Social Sciences Research, Bethesda, MD USA; 6 Department of Medicine, University of Washington, Seattle, WA USA; 7 Department of Epidemiology, University of Washington, Seattle, WA USA

**Keywords:** HIV, Long-acting antiretroviral, Acceptability, Feasibility, Kenya

## Abstract

**Background:**

In 2020, 14% of diagnosed persons living with HIV (PLWH) in Kenya were not taking antiretroviral therapy (ART), and 19% of those on ART had unsuppressed viral loads. Long-acting antiretroviral therapy (LA-ART) may increase viral suppression by promoting ART uptake and adherence. We conducted key informant (KI) interviews with HIV experts in Kenya to identify product and delivery attributes related to the acceptability and feasibility of providing LA-ART to PLWH in Kenya.

**Methods:**

Interviews were conducted via Zoom on potential LA-ART options including intra-muscular (IM) injections, subcutaneous (SC) injections, implants, and LA oral pills. KI were asked to discuss the products they were most and least excited about, as well as barriers and facilitators to LA-ART roll-out. In addition, they were asked about potential delivery locations for LA-ART products such as homes, pharmacies, and clinics. Interviews were recorded and transcribed, and data were analyzed using a combination of inductive and deductive coding.

**Results:**

Twelve KI (5 women, 7 men) participated between December 2021 and February 2022. Overall, participants reported that LA-ART would be acceptable and preferable to PLWH because of fatigue with daily oral pills. They viewed IM injections and LA oral pills as the most exciting options to ease pill burden and improve adherence. KI felt that populations who could benefit most were adolescents in boarding schools and stigmatized populations such as sex workers. SC injections and implants were less favored, as they would require new training initiatives for patients or healthcare workers on administration. In addition, SC injections would require refrigeration and needle disposal after use. Some KI thought patients, especially men, might worry that IM injections and implants would impact fertility, given their role in family planning. Pharmacies were perceived by most KI as suboptimal delivery locations; however, given ongoing work in Kenya to include pharmacies in antiretroviral delivery, they recommended asking patients their views.

**Conclusion:**

There is interest and support for LA-ART in Kenya, especially IM injections and LA oral pills. Identifying patient preferences for modes and delivery locations and addressing misconceptions about specific products as they become available will be important before wide-scale implementation.

## Background

Human immunodeficiency virus (HIV) remains a global pandemic. In 2020, the Joint United Nations Programme on HIV/AIDS (UNAIDS) estimated that there were 37.7 million people living with HIV (PLWH) globally. Approximately 87% of PLWH were on antiretroviral therapy (ART), of whom 90% were virally suppressed [1]. Sub-Saharan Africa remains the region most affected, accounting for 54% of PLWH globally [2]. In Kenya, the HIV prevalence among persons aged 15 to 49 years is 4.2%, with 86% of PLWH on ART and 81% of those on ART virally suppressed [1]. Adherence to daily oral treatment, which is required for viral suppression, is often compromised by factors such as forgetfulness, pill fatigue, and fear of social consequences such as stigma and discrimination. These challenges could undermine progress towards the UNAIDS 95-95-95 targets to eradicate HIV [3].

In well-resourced settings, long-acting cabotegravir-rilpivirine delivered monthly or every other month by intramuscular injection has been acceptable to patients and feasible when provided in diverse settings [3]. Additional long-acting antiretroviral therapy (LA-ART) regimens in development have been similarly acceptable and feasible in early phase trials [4]. These LA-ART regimens could improve adherence and increase treatment success by obviating the need to take a pill every day and travel with medications [5]. The extent to which novel LA-ART products can address adherence challenges globally, however, will ultimately depend on their acceptability to PLWH and the feasibility of their delivery in resource-limited settings. Sub-Saharan Africa, with the largest burden of HIV in the world, may have unique barriers and facilitators to the roll out and uptake of these products [6].

In our previous work, we investigated preferences for different LA-ART products, including intra-muscular (IM) injections, subcutaneous (SC) injections, implants, and LA pills, among PLWH engaged in care in Seattle, WA and Atlanta, GA, United States (US) [7–9]. This work was based on key informant (KI) interviews with US experts in HIV drug development or clinical research, which identified the LA-ART modalities most likely to become available in the near future [9]. In addition, these KI interviews identified product attributes such as injection site pain, location of administration, dosing frequency, and leeway in the event of a missed dose (which we called “late-dose leeway”) that were likely to influence product acceptability [9]. These attributes informed the development of a discrete choice experiment (DCE) that was successfully pilot tested and conducted in the US [7, 8].

The success of LA-ART in Kenya will depend on understanding patient preferences and any adaptations needed for delivery of the various options in a resource-limited setting [10]. We conducted KI interviews with HIV experts in Kenya to identify product and delivery attributes likely to be related to LA-ART acceptability among Kenyan PLWH and any program or service constraints that could influence feasibility and require adaptation.

## Methodology

### Study design

In this qualitative study, in-depth interviews were conducted with 12 Kenyan KI with expertise in HIV clinical care, peer education, research or policy. This work was done in preparation for adaptation and pilot testing of the DCE we had conducted in the US [8], prior to enrolling 700 PLWH in a DCE study to be conducted in Nairobi, Kenya.

### Recruitment and consenting

We identified KI through consultations with Kenyan and US-based study team members experienced in HIV research in Kenya, including individuals with connections to leadership in the Ministry of Health and several large treatment programs. Potential KI were contacted by phone and invited to participate via e-mail. Prior to the interviews, study procedures were explained to participants and written informed consent was obtained.

### Ethical oversight

Ethical approval was obtained from the Kenyatta National Hospital-University of Nairobi Ethics and Research committee and the University of Washington Institutional Review Board.

### Data collection

The US project leads (SMG, an HIV physician and clinical epidemiologist, and JMS, a clinical psychologist) conducted the interviews between December 2021 and February 2022. Interviews took place via Zoom, with recording to secure Cloud storage. A structured topic guide was used for the interviews, with follow-up probes to elicit additional information. Interviews were conducted in English and took approximately 45 to 60 min.

At the beginning of each interview, the purpose of the study was described and the different LA-ART modalities to be considered, including IM injections, SC injections, implants, and LA pills, were reviewed. In addition, we reviewed the assumptions, attributes and levels, and restrictions on specific combinations of attributes that we had used in the US DCE (Appendix 1). Following this review, KI were asked the following three questions:

1. Which treatment modality do you find most exciting and why?
2. Which treatment modality do you find least exciting and why?
3. What do you see as the challenges for providing combination LA-ART for HIV treatment in Kenya?

We then asked KI to reflect on any attributes (e.g., delivery site or dosing frequency) that could be challenging in Kenya and the reasonable levels (or range of levels) for each attribute in the Kenyan context. We specifically asked about considerations for ART-naïve (i.e., new to HIV treatment) vs. ART-experienced patients. In addition, we requested that KI point out any specific attributes or concepts the KI felt might be confusing or complicated for Kenyan patients to understand. We employed the “adaptome” framework for intervention adaptation to inform questions about important considerations for LA-ART roll-out in the Kenyan context [11]. This section of the topic guide included the following sections and questions from the adaptome framework [11].

1. Service setting adaptations: Who would deliver long-acting ART and in what clinical context? How would it be financed?
2. Target audience adaptations: Who do you think would be the target population for the different LA-ART modalities? Which patients would be most interested? Who would benefit most? What patient characteristics would be barriers to preference or use of specific treatment modalities?
3. Cultural adaptations: In what way might any of the treatment modalities need to be adapted or presented differently in the Kenyan context?

### Data analysis

Zoom-generated recordings were downloaded from the Cloud and saved to a password-protected drive. Audio recordings were transcribed through Otter.ai (audio-to-text transcription program), then reviewed and edited for accuracy by three team members (ATB, SMG, AK). Final edited transcripts were uploaded into the qualitative analysis software Dedoose (Hermosa Beach, CA, USA) for data management, coding and analysis. After agreement on an initial codebook based on the adaptome framework and other key concepts included in the topic guide, codes were applied by AK and CNM. Coding consistency between reviewers was assessed by JMS, and transcripts with discrepancies were discussed by the research team until resolution. The codebook was updated to incorporate inductive codes that emerged from the data as needed. Thematic analysis was used to identify themes and quotes related to the most and least exciting treatment modalities, the impact of the other six proposed DCE attributes on treatment preferences, and aspects of the Kenyan context that might influence feasibility and acceptability of the treatment modalities or require adaptations for LA-ART rollout.

## Results

### Participant characteristics

We interviewed 12 KI, 5 women and 7 men, aged 26 to 59 years. Their expertise was in oversight of HIV clinical care [3], peer education [2], school health [1], pharmaceutical research [1], sociobehavioral research [1], and policy making [4]. The median number of years of experience working in the HIV field was 12 (range: 7–20). Table 1 presents characteristics of the KI who participated. In the results below, participants were classified as clinicians, peer educators, researchers, or policy makers. Of note, the peer educators were PLWH who had received training to support other PLWH.

**Table 1:** Sociodemographic characteristics of the 12 KI

Continuous variables	Median (range)
Age (years)	41.5 (26–59)
Expertise working in HIV field (years)	12 [7–20]
Categorical variables	*N* (%)
Gender Female Male	5 (41.7) 7 (58.3)
Expertise HIV clinical care Policy makers Peer education* Pharmaceutical research Sociobehavioral research School health	3 (25.0) 4 (33.3) 2 (16.7) 1 (8.3) 1 (8.3) 1 (8.3)

* These two KI were PLWH

### Most preferred treatment modalities

Half of the KI (n = 6) reported long-acting oral pills would be the most preferred modality because they were painless and because PLWH had more experience with daily oral pills compared to other modalities. Additionally, KI thought these pills would be accessible and easily managed by providers (n = 2), and the reduced frequency of ingestion would improve adherence (n = 4).

*“I think that the long-acting orals will also be a good option for our clients. I mean, instead of a once-daily oral pill, they’re able to take a pill that does not require a frequency of a daily ingestion, to be able to suppress the virus, that could be quite a good option for our clients, if it becomes available…Especially for our clients [who] struggle with adherence.*” — Policymaker.

*“I’ll go with the long-acting oral pills… I think in terms of accessibility and the experience with using the oral antiretrovirals,… it will be easier to manage and…[acceptable] for the patients and… for their providers to handle something they are… used to, in terms of the tablet formulation, as opposed to the injectable antiretroviral formulations.”* — Clinician.

In contrast, some KI (n = 4) perceived LA IM injections to be the most exciting modality because they would reduce pill burden and make adherence much easier. Additionally, IM injections with longer dosing intervals (e.g., monthly or every 3 months) would be easily administered and convenient to PLWH (n = 4).

*“We have this injectable over here. And it’s much…easier, because… it will reduce the pill burden, and… this is one thing that you wouldn’t need to worry about carrying around.…With the pills, depending on the time one needs to take them, they’ll always need to be having them on their side.”* — Peer educator.

*“One of the reasons why I think the long-acting intramuscular injection is… convenient, it’s able to reduce the burden of the patient having to swallow pills every day… If you’re able to get like one injection for 3 months, that…eliminates the need to swallow tablets every day… Number two,…the reason I find it exciting is…we’ve struggled…with adherence of our clients… having to take pills on a daily basis… This…[simplifies the] modality of delivering…ARVs to the patient, and… eliminates that need to take pills daily,… and therefore could have better adherence compared to the oral medications… The main exciting thing about it is…the convenience,… the simplicity, and…the opportunity to improve adherence for our clients.” —* Policymaker.

### Least preferred treatment modalities

In contrast to the LA oral pills and IM injection, SC injections (*n* = 4) and implants (*n* = 5) were the least preferred. Perceived challenges were weekly dosing of SC injections (*n* = 5) and the increased number of required clinic visits if injections were not done at home (*n* = 3).

*“So if I have to inject every week, as opposed to, let’s say, taking an oral tablet, if that option is available every week, then I’ll opt to take the oral tablet every week, as opposed to the sub q injection.”* — Clinician.

*“Subcutaneous, I’m not so sure. It would be more frequent, I guess, than the intramuscular. So that should be in terms of when I’m thinking of patient visits, I’m thinking that would be difficult. Especially now that we have all these patients on differentiated care, and they are used to come in or seeking care twice a year. So this would mean that they’d be seeking care more frequently than normal.”* — Clinician.

In addition, several KI (*n* = 3) reported that PLWH who opt to use SC injections at home would need training on self-administration, injection site reactions, and needle disposal.

*“These patients… you have to give syringes to go home with. I don’t know whether…the sub-q, could that be given at the facility level? Or is the patient to be given another way to… like for diabetic patients, so we give the patient the insulin plus the needles and they do the sub-q injections at home? I think the sub-q is much lower in terms of my preference because of the cumbersomeness of the delivery or the delivery method of the sub-q injections.”* — Policymaker.

*“For [the] injection [under]… the skin, I think [we] would teach… the patient how to do it…How to take care of the injection site and…clean needles. This would be a lot…would need a lot of patient education”* — Clinician.

*Going with the first injection [subcutaneous], the one that is like the insulin injection…since one [does it alone]… training [will be required] on the proper use of a syringe…, [disposal] and [storage].”* — Peer educator.

While one KI thought that persons who inject drugs (PWID) would use the SC injection needles for recreational drug use and would not be comfortable issuing this modality, another felt that patients would experience stigma related to possessing needles and self-injection of medication, which are associated with PWID.

“*The sub-q injection… I’d have trepidations, especially for the injection drug users, when it comes to needles and giving them needles. And just, I probably most likely they… probably, you’re not sure of adherence, and then you’re also not sure what they’re going to be using their medicines for, you know. Give them that injection, I’d not be too keen on giving my PWID an injection. They probably want to experience some sort of high and shoot it up their veins or something, you know. So I wouldn’t give it to PWID”* — Clinician.

*“Definitely, there is a stigma, because the moment you’re seen with syringes, you’re associated with being a drug user…and it will be very hard. For example, people seeing syringes when they visit your home, or if someone opens your fridge and finds a bottle of vials for you to inject….”* — Researcher.

One KI reported that the subcutaneous injection may cause bruising, which would affect how women dressed.

*“Especially being a lady… at times we want to dress in tops that show a bit of our stomachs… when your stomach has bruises, it will be uncomfortable for you.”* — Peer educator.

Three KI predicted the uptake of implants would be low. Reasons given included that men might perceive implants as a women’s contraceptive and because implants are not a popular form of contraception among Kenyan women.

*“The association with family planning… maybe for the women because they already used to it in terms of the contraceptives… Maybe for the men, it might become a bit difficult to accept the implant.”* — Clinician.

*“The uptake of the implant for women’s contraception is really low as compared to other methods. So I don’t know how agreeable it would be for everyone… I know there’s going to be a lot of misinformation and apprehension in terms of using the implants.”* — Clinician.

Compared to other modalities, implants were also perceived by 3 KI to be problematic since they may lead to inadvertent HIV disclosure and stigma because of their visibility under the skin, especially because men could not ascribe this to having an implant for contraceptive purposes.

“*Maybe the implant for men might be a bit new. If you see a man with an implant, that is an ART [antiretroviral therapy] product. So… disclosure, could become an issue.”* —Researcher.

“*I think the implants…might be a bit of a problem, because I can see from the photos, it’s something that will be visible… An implant indirectly…discloses that someone is on antiretrovirals. So in terms of disclosure, that would be an issue.”* — Clinician.

Despite these perceptions, some KI (n = 3) thought that certain populations or target audiences would benefit from implants and injections because the longer dosing interval and reduced pill burden would improve adherence and reduce stigma. These KI felt that those who might benefit most would be men and adolescents who have challenges engaging in care.

“*I think my male patients would benefit from the implant… The length of time would be… okay… Usually, you’ll find that the men are missing more clinic appointments compared to their female counterparts*.” — Clinician.

“*Key populations would definitely be top of the target population, and adolescents and young youth, they don’t like taking pills, that is very common. Stigma [among] themselves is high. So imagine being seen with pills, for example, when you’re in a boarding school, and you have to take your 3-month supply of pills… So getting the quarterly injection beginning of the semester, and you wait for the other one, when you’re home. It would definitely increase their confidence… So many of the cases we’ve had of students, for example, in boarding school where the pills have been found, and it led to a lot of issues and stigma.”* — Researcher.

*“.Boarding school, is a little bit tricky.the matron is the one who keeps the drugs [ART]… or, maybe one [keeps their] own drugs [ART]…it’s a bit uncomfortable, or one is not confident to take the pill in front of.friends, or while they’re around school grounds. We had situations where we see kids who are… school-going tend to fail [virological failure], when they’re in school they are not adherent to the treatment [ART]…I know introducing the injectable or the implants will [benefit] them, because they wouldn’t be required to go to school carrying a bottle of pills, not knowing where to keep them, or hiding them somewhere so that friends will not see it while going through your stuff. So it’s going to be a big, big, big benefit.”* — Peer educator.

### Other Attributes

In our interview guide, we specifically queried about six attributes (Appendix 1) of the hypothetical product modalities that would be included in the planned DCE. Below we describe the main findings for each, how they might best be presented in the DCE, and how they might affect acceptability and feasibility.

According to 3 KI, the service setting or location where the LA product would be dispensed or administered was seen as likely to have a major impact on PLWH preferences. Factors such as burden to travel to a clinic and privacy concerns were raised.

*“In Kenya, we’re talking about clients who have come quite a long distance, I mean the distance from the clinic to home is quite a distance. So any option that keeps them away from the facility for as long as possible, they prefer to take it.*” — Policymaker.

Most KI (*n* = 9) considered pharmacies to be suboptimal delivery locations for LA-ART because it might be difficult for PLWH to disclose their HIV status and build rapport with the pharmacist. If included as an option, 3 KI felt that only licensed and registered pharmacies with regulatory oversight should offer LA-ART modalities.

*“Patients…are hesitant to disclose their status. So, this [pharmacy] is an extra facility that you have to disclose your status [to and]…start over again… So you need to build a relationship again with this facility….That is the challenge I foresee.”* — Clinician.

*“The issue of local pharmacy,…unlike [the] US,…a pharmacy could be one that is not run by a professional and it’s just a drug seller. But we’ve talked to a few people who think….we could [dispense ART in licensed pharmacies]… So we are interested in including it [as a location choice in the DCE], but it would have to be a qualified pharmacy that would be able to track the medications and probably have a registered pharmacist.”* — Policymaker.

Two KI in research and policymaking reported that while only facility-based pharmacies currently offer ART services, there is ongoing work in Kenya to include licensed, registered pharmacies within communities as alternatives for ART delivery. This work is important because pharmacies in the community are often the first point of contact for many people. In addition, local provision of ART could reduce the burden of travel to clinical sites.

*“….one of the things that we actually just learned from Nigeria where they implemented*.

*what they’re calling the “central distribution” of ART through pharmacies… community pharmacies. So in Kenya, we have not yet done this…but now that we are thinking about it, we are trying to put a policy framework to allow us to be able to deliver ART through pharmacies……within the hospital [or] in the clinic….and in town or somewhere, where a patient can be able to…access their ART…. We don’t have that option…or… model in Kenya, where our clients are able to access their treatment from a pharmacy…outside the facility. But it’s something that in another 1 or 2 years’ time, we are planning to put in place…*”– Researcher.

*“As a country,…we want to look at community pharmacies, like retail pharmacies, as another avenue for people accessing drugs. So traditionally, in our HIV programme, all our service delivery modes have been around hospitals, clinics and mission hospitals. And we haven’t utilized…retail pharmacies. And they’re quite many, and they are the ones who serve a lot of the communities because they tend to be the first drop-off place for clients. So maybe by the time this comes, our plans for seeing how registered pharmacists can be an alternative for that connection would already be in process. And therefore it would be easy to have that conversation. We have the DSD [differentiated service delivery] discussions that have been ongoing, and therefore even among the key expert clients, they know that that is something that is in progress.”* — Policy Maker.

Not surprisingly, many KI (two clinicians, two policy makers and a researcher) thought that frequency of dosing was an important consideration both for PLWH and for clinic staff. Less frequent dosing, especially if dosing required a clinic visit, was preferred over more frequent injections.

*“I think…what would make it agreeable would be the length of time… I have something that I don’t have to come to the clinic for… I don’t have to take drugs for a year… I think that would be agreeable. It would be good to hear what the patients say about it.”* — Clinician.

*“Long-acting orals, my feel is, it just depends on the interval. Weekly, I’m not so sure. If it’s longer than that, probably more patients may opt, especially those patients who are on multiple drugs, those who have other comorbidities, and they’re on multiple pills. So this will be an option to basically reduce their pill burden.*” — Clinician.

On the other hand, one KI felt that less frequent dosing would keep the patients away from the health facility, which would interfere with their viral load monitoring and psychosocial support.

*“Implants [which] last 12 months, you can’t be away [for]12 months. That’s…too long. But maybe 6 months? Yeah, you might still have a 6 month viral check, just to see how it’s going or maybe even just a psychosocial support check.” —* Researcher.

While several KI (*n* = 4) felt that pain would be a barrier for some clients, other KI (*n* = 3) felt that PLWH would be able tolerate pain, given the mitigating effects of longer dosing intervals and if convenient locations could be used.

*“If the pain is moderate, but the frequency is longer, and the location is very near, the pain would not be a problem, even if the pain is… moderate.”* — Clinician.

Feedback on the other attributes primarily focused on clarifying the terms for future DCE participants. Five KI concurred that pretreatment time undetectable might be more of a concern for clinicians and thought the term would not be understood by PLWH. They recommended the use of the term “undetectable viral load” or “suppressed” in presenting this concept to DCE participants.

*“Probably in the pre-treatment time undetectable, we may need to make it very….[clear]. that should be the viral load undetectable.”* — Policymaker.

Two KI also cautioned that we should be careful in defining what we meant by the term late-dose leeway for DCE participants. Some KI (n = 4) felt that this attribute should not be included in the DCE because PLWH would not adhere to the recommended dosing intervals if they were told doses could be taken late.

“. *I think it [is] important information for the clinician to have. It’s good if we tell the patients that this drug is taken every 4 weeks, and they do try as much as possible to come every 4 weeks. And, we leave it there. Maybe if they ask about leeway, maybe we can say okay, with… maybe a day or two. I think at the back of my mind, I know you have a week’s leeway. But I really don’t want to, because, knowing our patients, they will stretch it and they will come on the last day.*” — Clinician.

Three KI thought the term negative reaction testing would be confusing to PLWH and recommended simpler terms such as “allergy” or “side effect” to explain this attribute to DCE participants. These simpler terms would also help address their concerns and fears when introducing the long-acting options.

*“For the adverse [reactions],… I think that this is a bit complex, we need to find a way of simplifying it… Sometimes introducing adverse reactions… may help address their concerns, but sometimes also it may scare somebody. So, how you introduce it is also very important.…So, we start with one that is not so long-acting, just to see if you’re not allergic. If you’re not allergic, then we switch to the long-acting. I think that will simplify it. That’s, much, much better in my opinion.”* — Clinician.

### Adaptations for the Kenyan context

Most KI (n = 7) noted factors related to the economic, social, and cultural context in Kenya that might affect the acceptability of various LA-ART modalities and feasibility of delivery and longer-term, sustained implementation in the country, given limited resources. For example, service setting challenges that KI thought could impact LA-ART rollout included limited space to administer injections in privacy, the need to train healthcare providers to insert implants, increased workload for healthcare providers (e.g. implant insertion), and the need to hire more healthcare providers if clinic visits by PLWH increased due to injections.

“*Other challenges I’m also foreseeing include: we may need to include increased resources to deliver these treatment modalities. And the reason I am saying is: currently, most of our patients are on either 3-month or 6-month drug refills. So their [clinic] visits, you will find, are less frequent, especially those on 6-month refills. So if you have to come to the [clinic] to be injected every 2 months or every month… So you see, the frequency of [clinic] visits will increase. We’ll need more staff to administer. Probably, I’m looking at also the space to administer these injections. If you have to put an implant of course, again, it also comes with its own requirements. So those are some of the challenges that I do foresee*.” — Clinician.

*“You’d need a skill, you know, the current situation is that we have maybe for the pharmacy, personnel dispensing the oral medications, but you know, even in the planning setup it’s the nurses who do the contraceptives. So I don’t know, I think you need….to train people, especially nurses on how to do the implants.”* — Clinician.

Several KI (*n* = 5) felt that more frequent appointments and travel to the clinic for injections by individuals would be challenging for patients who were used to clinic visits every 4 to 6 months in Kenya’s differentiated care model. This could especially be the case for individuals who live far from the clinic.

“*We’d struggle with clients… used to having…a 90-day pack [or even] have two of them,…[they] don’t need to come to the facility up to 6 months…The option… [of] a monthly injection…and [one has] to come to the facility… that could be something that could potentially be a barrier”* — Clinician.

Four KI (two clinicians, a policy maker and a researcher) foresaw challenges with LA-ART requiring cold chain storage because many patients would not have access to refrigerators.

*“I’d be thinking in terms of socioeconomic [barriers]… Like, do you have a fridge? Are you able to store this? You know… that’s what I’d be thinking for the sub-q.”* — Clinician.

*“When we had the cold storage products…[that] necessitated the ones who could not access…a fridge, [to] make rapport with their local chemist, shop, or their…neighbor to keep for them those drugs.*” — Policymaker.

Additionally, seven KI (three clinicians, two policy makers and two researchers) noted that once LA-ART was rolled out, they would need to ensure availability and accessibility by PLWH, in settings where consistent supply has previously been a challenge. Further, having fair criteria to determine who should receive LA-ART if supply were limited would be important. Therefore, the target audience for this innovation required careful reflection.

*“I mean, is it something that will be available for everyone? Who do we decide.[should] get it? What reason do we give those who are not getting it?… So I mean, those are the kind of barriers that would be there… There [are].challenges with supply chains that will probably be one [of].the barriers. So can we ensure consistent supply?”* — Clinician.

Regarding cultural adaptations, three KI felt it would be important to teach PLWH and providers about LA-ART prior to rollout, in order to alleviate their concerns about safety and drug interactions and debunk the myths and misinformation Kenyan PLWH may have.

“*One big challenge I’ve actually seen in Kenya, when you’re rolling out a new health product, is people tend to worry about the safety profile of the drug. People tend to worry about the side effects of the drug. For some reason, people tend to worry about fertility. If it’s going to affect their fertility, normally, is one of the top concerns that most people do have. I’m thinking especially of long-acting injectables and implants, because these are methods that we have been using to deliver contraceptives.”* — Clinician.

“*…. And what happens when you can’t remove it from the body? So once you have already administered and then maybe adverse effects, your comorbid conditions? Or maybe there are some drug interactions. I don’t know how you’d have to deal with that. Because for the once daily, then it’s possible to just stop and then maybe change to something else or some other form of treatment.”* — Clinician.

*“…Working on marketing and giving proper information… those who are illiterate…[can also].understand. If it’s marketed well, majority…will [use] the new injectable, as compared to the old pill” —* Peer educator.

## Discussion

In this qualitative study of potential acceptability of LA-ART in Kenya, KI thought the most exciting modalities were LA oral pills and IM injections, while the least exciting modalities were SC injections and implants. They felt that product characteristics such as location, pain, and frequency would be predictive of LA-ART acceptability. In contrast, more complicated concepts such as the attributes we labeled pretreatment time undetectable, negative reaction testing, and late-dose leeway (Appendix 1) would need to be carefully described to be understood. They also reported socioeconomic and cultural factors might affect the acceptability of different LA-ART modalities and the feasibility of their delivery and sustained implementation in Kenya.

These findings are consistent with previous studies conducted in sub-Saharan Africa and other resource-limited settings among potential LA-ART users, which showed good acceptability of IM injections because of their longer dosing frequency, reductions in pill burden and reductions in stigma [12, 13]. Although LA IM injections have some disadvantages, such as pain and injection site reactions, many patients value decreased pill burden and improved adherence more and are not deterred [12, 13]. For instance, a qualitative study conducted by Simoni et al. among a diverse population of adults and youth living with HIV in coastal Kenya found that participants felt injections would alleviate the burden of daily pill-taking while avoiding inadvertent disclosure and HIV stigma [6]. Further, a study conducted by Tosca et al. in South Africa among adolescents and youth living with HIV found six factors associated with preference for injectable LA-ART: medication stock-outs, experiencing side-effects, pill-burden, past-year treatment changes, any HIV stigma and recent ART initiation [12]. In sub-Saharan Africa, evidence suggests that frequent visits to HIV clinics are a barrier for retention in HIV care [14] which could cause problems for any LA-ART modality dosed in clinic if visit frequency would increase for a given patient.

Of note, SC injections were among the least preferred LA-ART modalities in our study. In the material provided to KI, attributes for SC injection were restricted to the following based on feedback from our US KI: no or mild pain; administration at home, clinic or pharmacy; and frequency of 1, 4, 8 or 12 weeks [9]. Given this information, our Kenyan KI were concerned that SC injections would require frequent clinic visits, training of healthcare workers if delivered in clinic, training of PLWH, refrigeration, and steps to address stigma if used at home. Challenges with insulin injections for diabetics came up frequently when discussing SC injections. The use of SC insulin injections for diabetes care is negatively impacted by the need to educate patients and train them on injection techniques [15, 16] and the requirement for refrigeration or cooling below room temperature during transportation and storage [16]. These challenges and affordability issues likely undermine adherence to insulin therapy and limit the availability of diabetic medication in African settings [17, 18]. In addition, the KI were concerned about stigma related to having injections equipment at home; similar social stigma has been reported among diabetic patients who required SC insulin injections in Malaysia [15] and among users of depot-medroxyprogesterone acetate (DMPA) contraception for home injection in Malawi [19]. Thus, equipment such as injector pens that hide the needle for injection [15] and may decrease visible bruising [20] should be considered if SC LA-ART is rolled out in Kenya or similar settings. Other challenges with SC will also need to be addressed.

Implants were also not strongly favored. KI noted that visibility under the skin, which may lead to inadvertent HIV status disclosure, and their association with female contraception, which could lead to stigma especially among men. If implants become available for LA-ART in Kenya and similar settings, consideration should be given to inserting implants into bodily sites not readily visible to others, especially for men, or designing implants that are less visible after insertion. In addition, KI thought required training for healthcare providers to insert implants safely would add burden on the health system and likely increase provider workload. Interestingly, a study of contraceptive implant use in Africa reported improved uptake and acceptability of contraceptive implants over time (including in Kenya, which had the highest implant contraceptive prevalence rate in the world at the time of that study, at 18.1%) because they were less demanding to the healthcare system than quarterly DMPA injections and could potentially be inserted by trained providers, including frontline and community workers [21]. This indicates that implants may be more promising than judged by many of our KI. In fact, several KI thought implants could be advantageous to certain populations such as adolescents, who struggle to take their medication and attend clinic visits, especially if in boarding schools.

KI in this study provided important feedback on how to present the attributes and levels for our planned DCE conducted with Kenyan PLWH. They emphasized the need to provide simple instructions and carefully word more complex attributes, such as pretreatment time undetectable, negative reaction testing, and late-dose leeway. Interestingly, one KI felt that Kenyan PLWH would take the concept of “late-dose leeway” as permission for delaying their doses or clinic visits, suggesting that this attribute could be more important in Kenya. In the DCE conducted among PLWH in the US, modality, frequency, and pain were by far the most important attributes influencing preferences, followed by delivery location, and minimal impact for the more complex attributes [8]. Findings of our current study suggest that patient preferences may follow a similar pattern in Kenya as they did in the US [8]. Regardless of final results, the KI interviews provided important information that will inform adaptation of DCE to the Kenyan context for pilot testing, which will provide additional feedback to guide adjustments as needed, similar to our pilot testing for our DCE in the US [22].

Feedback from KI also brought out concerns about challenges with the equitable delivery and widespread use of LA-ART in Kenya, including the need for service setting, target audience, and cultural adaptation of programming. These included ensuring ongoing availability and broad accessibility, identifying appropriate candidates for LA-ART should supplies be limited, addressing provider and patient concerns about safety and drug interactions, and ensuring that the health system is adequately staffed and supported for frequent injection appointments and for cold chain requirements for storage, if needed. Some KI provided feedback related to decentralization of ART services to the community and consideration of pharmacy-based delivery during roll out of LA-ART, as previously recommended in low and middle income countries to maximize HIV mangement [23]. The need to prioritize patients who would benefit most from LA-ART if supplies were limited has been a topic of discussion in the US for injectable LA-ART [24] and merits further research and discussion for LA-ART rollout in sub-Saharan Africa.

This study had several strengths, including a focus on more than one LA-ART modality and recruitment of a diverse group of KI with expertise in HIV policy, clinical care, research, and peer support. As such, it adds much needed data on the potential acceptability and feasibility of LA-ART delivery in African settings. Study limitations included the small number of KI interviewed in each category; however, they constituted a diverse group that brought different perspectives from their expertise, resulting in good information power [25]. In addition, these results cannot be extrapolated to real-world experience, because LA-ART are not yet available in Kenya. However, these findings will help inform future policy during scale-up and implementation as LA-ART products when different products and modalities become available.

In conclusion, KI in Kenya were excited about LA-ART regimens with longer dosing intervals, especially long-acting tablets and IM injections, while those that required training of patients or providers or could lead to stigmatization, such as SC injections and implants, were the least exciting. Locations that were regulated and had adequate privacy were preferred for delivery. While the feasibility of LA-ART delivery in Kenya will be impacted by a number of considerations, further research on patient preferences for LA-ART will help inform the eventual wide-scale implementation of the most promising LA-ART products in Kenya and similar settings.

## Data Availability

The datasets used and/or analysed during the current study are available from the corresponding author on reasonable request.

## Appendix 1: information provided to key informants about hypothetical LA-ART products

### Assumptions

- All products to treat HIV infection that will reach the market will result in viral suppression if taken as prescribed, and no products will lead to a cure.
- Assume no difference in out-of-pocket costs for these medications, compared to your current regimen.
- Assume that clinic follow-up for monitoring will be similar regardless of product type.
- Side effects other than injection site pain or reactions are determined by the specific drugs in the regimen or product, and not by the route of administration. Therefore, we will not ask about specific side effects other than injection site-related.

### Attributes and Levels

**Table:**

Attribute	Levels
Treatment type	Injection into muscle, injection into the skin, implant, oral tablet vs. status quo (daily oral)
Location of administration	Home, clinic, pharmacy
Frequency of dosing	1 week, 1 month, 2 months, 3 months, 6 months, 12 months
Pain	None, mild, moderate
Pre-treatment time undetectable	None, 3 months, 6 months
Pre-treatment negative reaction testing	Needed, not needed
Late dose leeway	Short period, Long period
**Product Descriptions**	
Constant comparison: Patient’s current daily oral regimen.	
Injection into muscle: 1-, 2-, or 3-month frequency; clinic or pharmacy location; mild or moderate injection site pain/reaction.	
Injection into skin: 1 week or 1-, 2-, or 3-month frequency; any location; none or mild injection site pain/reaction. If frequency = 1 week then location = home.	
Implant: 6 or 12 months; only clinic; mild or moderate insertion site pain/reaction. Side effects quickly reversible by removal.	
Long-acting oral: 1 or 4 weeks; only home; no injection site pain/reaction.	

## Declarations

This material should not be interpreted as representing the viewpoint of the U.S. Department of Health and Human Services, the National Institutes of Health, or the Office of Behavioral and Social Sciences Research.

## Abbreviations

ART: Antiretroviral treatment
DCE: Discreet Choice Experiment
DMPA: depot–medroxyprogesterone acetate
HIV: Human Immunodeficiency Virus
IM: intramuscular
KI: Key informants
LA: Long acting
PLWH: People living with HIV
PWID: Persons who inject drugs
SC: Subcutaneous
US: United States
